# Supplementary material for: Hydrophilic nanoparticles that kill bacteria while sparing mammalian cells reveal the antibiotic role of nanostructures
Source: Nat Commun. 2022 Jan 11;13:197. doi: 10.1038/s41467-021-27193-9 (PMC8752835; doi:10.1038/s41467-021-27193-9)
Supplement: Supplementary file 1 — Supplementary Information [file 41467_2021_27193_MOESM1_ESM.pdf]

Supplemental Information

Hydrophilic Nanoparticles That Kill Bacteria While Sparing Mammalian Cells Reveal the Antibiotic Role of Nanostructures

Yunjiang Jiang, Wan Zheng, Keith Tran, Elizabeth Kamilar, Jitender Bariwal, Hairong Ma, Hongjun Liang*

*: H.liang@ttuhsc.edu

Table of Contents

1. Synthesis of model NPPBs 2

1.1 Synthesis of well-defined silica nanospheres 2

1.2 Synthesis of ATRP macroinitiator SiO_2@Br 3

Figure 1. Synthesis of ATRP macroinitiator on small silica nanospheres (d≤50 nm). 3

Figure 2. Synthesis of ATRP macroinitiator on large silica nanospheres (d>50 nm). 4

1.3 Synthesis of free ATRP initiator 2-bromo-2-methyl-N-hexyl propanamide (C_6-Br). 4

Figure 3. Synthesis of ATRP initiator C_6-Br. 4

1.4 Synthesis of model NPPBs and free P4VP grown simultaneously with SiO_2@P4VP 5

Figure 4. Synthesis of model NPPBs with free P4VP grown simultaneously. 5

2 Cleavage of brush polymers from SiO_2@P4VP 6

3 Structural characterization and biological activities of model NPPBs 6

Figure 5. The successful synthesis of model NPPBs as characterized by FT-IR. 7

Figure 6. The well-defined polymer brushes on model SiO_2@P4VP as characterized by NMR and GPC. 8

Figure 7. TGA of silica nanospheres before and after SI-ATRP of polymer brushes to prepare model NPPBs. 9

Figure 8. Zeta potentials of model NPPBs. 10

Figure 9. Bare silica nanopsheres without polymer brushes show no antimicrobial activity or cytotoxicity. 11

Figure 10. MBC assays of model NPPBs reveal nanoparticle size-dependent bactericidal activity. 12

Figure 11. Snapshots examples of dye leakage assays that reveal NPPBs remodel bacteria- and mammalian cell-mimicking GUVs in different modes depending on both the intrinsic curvature of membrane lipids and nanoparticle size. 13

Figure 12. The targeting and disruption of bacterial membranes by NPPBs is independent on bacterial membrane potential. 14

Figure 13. SAXS reveals that the formation of pores in model membranes encountered with small NPPBs (d_{silica}≤50 nm) depends highly on their content of negative-intrinsic-curvature lipid. 15

4 References 16

1. Synthesis of model NPPBs

1.1 Synthesis of well-defined silica nanospheres

Synthesis of S7, S10, and S25. Very small silica nanospheres (d<30 nm) can be synthesized by sol-gel reactions in the presence of amino acids.[1-3] The slow hydrolysis of TEOS catalyzed by the basic amino acids, such as lysine and arginine, leads to the formation of mono-dispersed silica nanospheres with controllable sizes depending on the reaction stoichiometry of precursors. For example, S10 was synthesized by stirring a mixture of TEOS (5.21 g, 25 mmol), L-lysine (73.1 mg, 0.5 mmol) and water (72.9 g, 4.05 mol) at room temperature for 48 h, followed by incubating at 100 °C for another 24 h, and the final product was purified by dialysis in water for 2 days (with MWCO 12 kDa membrane). Ethylene glycol (20 mL) was then added and water was removed by the rotary evaporator at 80 °C. By varying the ratio of TEOS, L-lysine and water, silica nanospheres of different sizes can be synthesized.[1-3]

Synthesis of S50. S50 was synthesized by seeded-growth method as reported.[4] In a typical run, S25 was first synthesized by arginine catalyzed sol-gel reaction. A mixture of L-arginine (91 mg, 0.52 mmol), cyclohexane (4.5 mL), water (69 g, 3.83 mol) and TEOS (5.21 g, 25 mmol) was maintained at 60 °C and stirred for 24 h. The cyclohexane was evaporated by an air flow. S50 was later synthesized using the resultant S25 as seeds. Briefly, S25 solution (15 mL) was diluted with water (54 mL) so that the final arginine concentration was in the range of 1-2 mM. Subsequently, Cyclohexanes (7.5 mL) and TEOS (4.96 g, 23.8 mmol) were added and the volume ratio of total TEOS to cyclohexane was adjusted to be slightly below 1:1. The mixture was maintained at 60 °C and stirred for 24 h. After the hydrothermal treatment at 100 °C for 24 h, S50 was purified by dialysis. The solvent was then replaced by ethylene glycol and water was removed.

Synthesis of S110 and S270. Large silica nanoparticles (d>50 nm) can be conveniently synthesized by the Stöber method.[5, 6] The procedures for the synthesis of S110 and S270 are similar and only differ by the feed ratio of reagents. Taking the synthesis of S270 as an example, TEOS (6.26 g, 30 mmol) was added dropwise into a mixture of water (11.3 g, 0.62 mol), ammonium hydroxide (29% NH_3, 1.82 g, 31.0 mmol NH_3) and ethanol (63.1 g, 1.372 mol). The number ratio of TEOS:NH_3:H_2O:ethanol was set as 30:31:620:1372. The mixture was stirred at room temperature for 18 h. The S270 product was then collected by centrifuge at 10,000 x g for 10 min and washed by ethanol twice. A similar procedure was used for the synthesis of S110, except that the number ratio of TEOS:NH_3:H_2O:ethanol was changed to 40:15.5:56:1420.

1.2 Synthesis of ATRP macroinitiator SiO_2@Br

Synthesis of S7@Br, S10@Br, S25@Br, and S50@Br. As shown in Figure 1, 2-bromo-2-methyl-N-(3-(triethoxysilyl)propyl) propanamide (APTES-Br) was first synthesized by reacting APTES with α-bromoisobutyryl bromide (BIBB).[7]

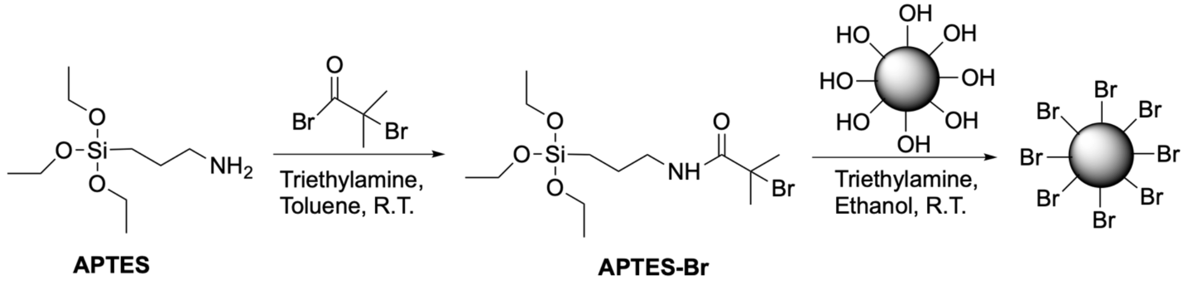

Figure 1. Synthesis of ATRP macroinitiator on small silica nanospheres (d⩽50 nm).

Briefly, APTES (2.21 g, 10 mmol) and triethylamine (1.01 g, 10 mmol) were dissolved in anhydrous toluene (70 mL) and added into a flask equipped with a stirring bar. BIBB (2.6 g, 11.3 mmol) in anhydrous toluene (30 mL) was added dropwise into the mixture. The mixture was allowed to stir at 0 °C for 3 h and then at room temperature for 12 h. The insoluble solid was removed by centrifugation, and the solvent was removed under reduced pressure. The product was re-dissolved in anhydrous dichloromethane and the insoluble solid was removed by filtration. After removing the solvent again under reduced pressure, the product was dried in vacuum and the successful synthesis was confirmed by ^{1}H NMR ($CDCl_3$): δ 3.82 (5H, -Si-(OC**H**$_2$CH$_3$)$_3$), δ 3.27 (2H, -C**H**$_2$NH-), δ 1.94 (6H, -C(C**H**$_3$)$_2$Br), δ 1.65 (2H, -C**H**$_2$CH$_2$NH-), δ 1.22(8H, -Si-(OCH$_2$C**H**$_3$)$_3$), δ 0.63 (2H, Si-C**H**$_2$CH$_2$-).

The macroinitiators (i.e., SiO_2@Br) were prepared by reacting the Si-OH groups on the surface of silica nanospheres with APTES-Br.[8] Silica nanosphere solution in ethylene glycol (7 mL, ~0.5 g silica nanoparticles) was dried in vacuum oven to remove trace amount of water. Triethylamine (1.5 g, 14.8 mmol) and anhydrous ethanol (84 mL) were added to the silica nanosphere solution. APTES-Br (1.5 g, 4 mmol) in anhydrous ethanol (6 mL) was added dropwise to the mixture. The mixture was stirred at room temperature for 24 h. Solvent was removed by rotary evaporator and the product was precipitated into a hexanes/ether mixture (1/1 volume ratio) twice. The final product was collected by centrifugation and dried in vacuum.

Synthesis of S110@Br and S270@Br. We found that the abovementioned method works ineffectively for large silica nanospheres (d>50 nm). To improve on that, we adapted a different

approach as shown in Figure 2 instead. Briefly, S110 and S270 were surface-modified with ATPES first,[9] then reacted with BIBB via an acylation reaction.

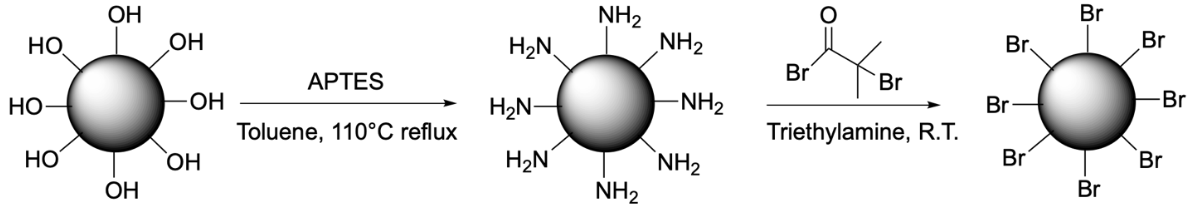

Figure 2. Synthesis of ATRP macroinitiator on large silica nanospheres (d>50 nm).

Taking the synthesis of S270@Br as an example, S270 (400 mg, ~0.03 mmol -OH) was dispersed in anhydrous toluene (100 mL) in a flask equipped with a stirring bar. APTES (0.322 g, 1.5 mmol) was added into the suspension and the mixture was then reflux at 110 °C for 24 h. The nanoparticles were collected by centrifuge and washed by ethanol three times. After solvent removal, S270@NH_2 (400 mg, ~0.03 mmol –NH_2) was re-dispersed in anhydrous CH_2Cl_2 (40 mL) containing triethylamine (0.4 g, 4.0 mmol). BIBB (1.86 g, 8.1 mmol) dissolved in anhydrous CH_2Cl_2 (10 mL) was added dropwise to the nanoparticle dispersion. The mixture was stirred in ice bath for 3 h, followed by stirring at room temperature for 10 h. The final products were then collected by centrifugation (7,000 x g, 5 min) and washed with ethanol/water = 1/1 twice, and then with pure ethanol twice. Solvent was removed in vacuum.

1.3 Synthesis of free ATRP initiator 2-bromo-2-methyl-N-hexyl propanamide (C_6-Br).

It has been well recognized that in the synthesis of polymer brushes on nanoparticles via surface-initiated controlled/"living" polymerization, little difference exists between the polymer brushes grown on the nanoparticles and free polymers grown simultaneously in the reaction batch.[8-12] To aid the characterization of the polymer brushes, we synthesized a free ATRP initiator C_6-Br that has a very similar chemical structure as the surface-bound ATRP initiator (Figure 3).

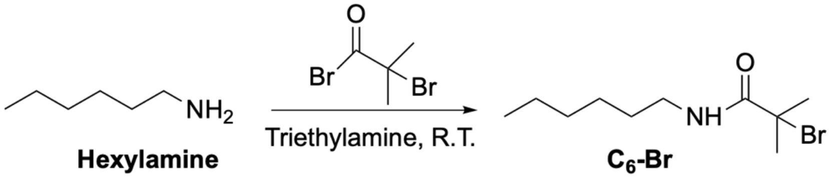

Figure 3. Synthesis of ATRP initiator C_6-Br.

Briefly, Hexylamine (1.0 g, 9.9 mmol) and triethylamine (2.0 g, 19.8 mmol) were dissolved in anhydrous dichloromethane (20 mL) and added into a flask equipped with a stirring bar. BIBB

(4.55 g, 19.8 mmol) in anhydrous dichloromethane (10 mL) was added drop-wise into the mixture. The mixture was kept at 0 °C for 2 h and then at room temperature for another 10 h. The insoluble salt was removed by filtration and the solution was washed sequentially with 5% $NaHCO_3$ solution (3x100 mL), 0.5 M HCl solution (3x100 mL), and saturated NaCl solution (3 x 100 mL). Water in the suspension was removed by $MgSO_4$ and the organic solvent was removed by rotary evaporator and vacuum. A light-yellow liquid product was obtained and the successful synthesis was confirmed by ^{1}H NMR. ^{1}H NMR ($CDCl_3$): δ 3.23 (2H, -C**H**$_2$NH-), δ 1.93 (6H, -C(C**H**$_3$)$_2$Br), δ 1.53 (2H, -C**H**$_2$CH$_2$NH-), δ 1.29 (6H, CH$_3$-C**H**$_2$C**H**$_2$C**H**$_2$-), δ 0.87 (3H, C**H**$_3$-CH$_2$CH$_2$CH$_2$-).

1.4 Synthesis of model NPPBs and free P4VP grown simultaneously with SiO_2@P4VP

As shown in Figure 4, the SiO_2@P4VP was first synthesized by SI-ATRP of 4-VP with SiO_2@Br as the macroinitiator and Me$_6$TREN/Cu(I)/Cu(II) as the catalyst. Free P4VP was also grown in the same reaction batch using C_6-Br as the free initiator.

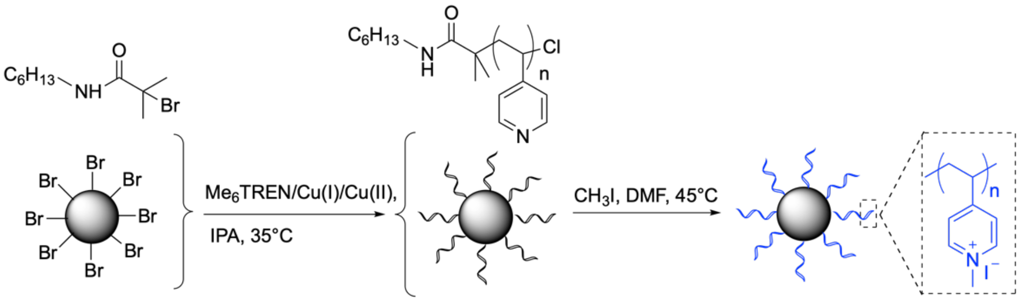

Figure 4. Synthesis of model NPPBs with free P4VP grown simultaneously.

Using the synthesis of S10-P33 as an example, a mixture of S10-Br (40 mg/mL in DMF, 2 mL, 80 mg, ~0.12 mmol -Br), C_6-Br (22.6 mg, 0.09 mmol), 4-VP (2.39 g, 22.8 mmol), $CuCl_2$ (10.0 mg, 0.074 mmol), Me$_6$TREN (17.0 mg, 0.074 mmol) dissolved in 3.8 mL isopropyl alcohol (IPA) was added into a 10 mL Schlenk flask equipped with a magnetic stirring bar. We used the graft density of the ATRP initiator as determined previously (~ 4/nm^2)[12] in the reaction design. After degassed by two freeze-pump-thaw cycles, a mixture of CuCl (7.3 mg, 0.074 mmol) and Me$_6$TREN (17.0 mg, 0.074 mmol) dissolved in IPA (1.3 mL) was injected to the solution under a nitrogen flow. The flask was sealed after another three freeze-pump-thaw cycles and immersed in a water bath at 35 °C for predetermined times based on the kinetics study. In order to minimize cross-linking and radical-radical coupling termination, the polymer conversion was controlled to be less than ~15%.[13] The SiO_2@P4VP product was collected by precipitating in 10-fold acetone twice, dried

in vacuum, and quaternized by a reaction with methyl iodide to turn the P4VP brushed into hydrophilic and cationic P4MVP.[12] On the other hand, the free P4VP in the same reaction batch was recovered from the acetone supernatant by precipitating the concentrated solution residuals into excess hexane twice. The solid was dried in vacuum. The Me_6TREN/Cu(I)/Cu(II) complex in free polymer was then removed by passing the polymer solution dissolved in CH_2Cl_2 through a column filled with neutral Al_2O_3. The purified free P4VP was recovered again by precipitation.

2 Cleavage of brush polymers from SiO_2@P4VP

We also compared the molecular weight and molecular weight distribution of P4VP brushes with that of the free P4VP grown simultaneously with SiO_2@P4VP by cleaving the P4VP brushes via HF digestion.

Taking the cleavage of S10@P4VP as an example, S10@P4VP (0.1 g, ~50% P4VP) and tetrabutylammonium bromide (5 mg, 0.015 mmol) were dispersed in CH_2Cl_2 (10 mL) and added into a 50-mL PTFE container. HF (0.5 mL, 48% HF) was added into the mixture under vigorous stirring. The mixture was stirred for 4 h and the solvent was evaporated in the fume hood. A mixture (5 mL) of DMF with 1 M NaOH was added to re-dissolve the polymer. The solution was dialyzed (with MWCO 2000 Da membrane) in alkaline water (pH≥11) for 2 days and then in Millipore water for another 2 days. The polymer was then collected by centrifuging and dried in vacuum. Note that if the P4VP cannot be fully dissolved in CH_2Cl_2, it indicates a portion of the P4VP will be still in salt form. More alkalization will help neutralize the residue HF from P4VP.

3 Structural characterization and biological activities of model NPPBs

We used a plethora of characterization methods to confirm the successful synthesis of model NPPBs with well-defined structures. Some of the structural characterization data and biological assays not presented in the main text are included here.

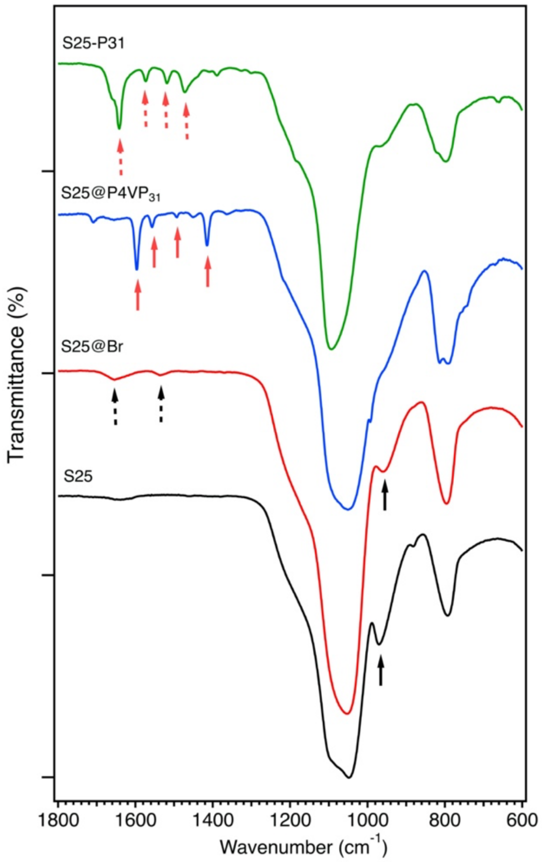

Figure 5. The successful synthesis of model NPPBs as characterized by FT-IR.
As an example, FT-IR spectrum of S25 (black), S25@Br (red), S25@P4VP$_{31}$ (blue) and S25-P31 (green trace) are compared. The Si-OH stretching at 950 cm^{-1} (marked by solid black arrows) is drastically weakened once S25 is converted to S25@Br, which is accompanied by the appearance of two new bands at 1650 cm^{-1} and 1550 cm^{-1} (marked by dotted black arrows), respectively, corresponding to the amide I and amide II bands from APTES-Br. After SI-ATRP of S25@Br to synthesize S25@P4VP$_{31}$, 4 characteristic bands of the pyridine moieties at 1595 cm^{-1}, 1554 cm^{-1}, 1494 cm^{-1} and 1414 cm^{-1} (marked by solid red arrows), respectively, show up clearly. The successful quaternization reaction that turns S25@P4VP$_{31}$ into the final hydrophilic NPPB (i.e., S25-P31) is confirmed by the complete shift of the 4 characteristic pyridine peaks to higher wavenumbers at 1639 cm^{-1}, 1570 cm^{-1}, 1515 cm^{-1}, and 1468 cm^{-1}, respectively (marked by dotted red arrows), suggesting 100% degree of quaternization. This same method was used to characterize the successful synthesis of all model NPPBs.

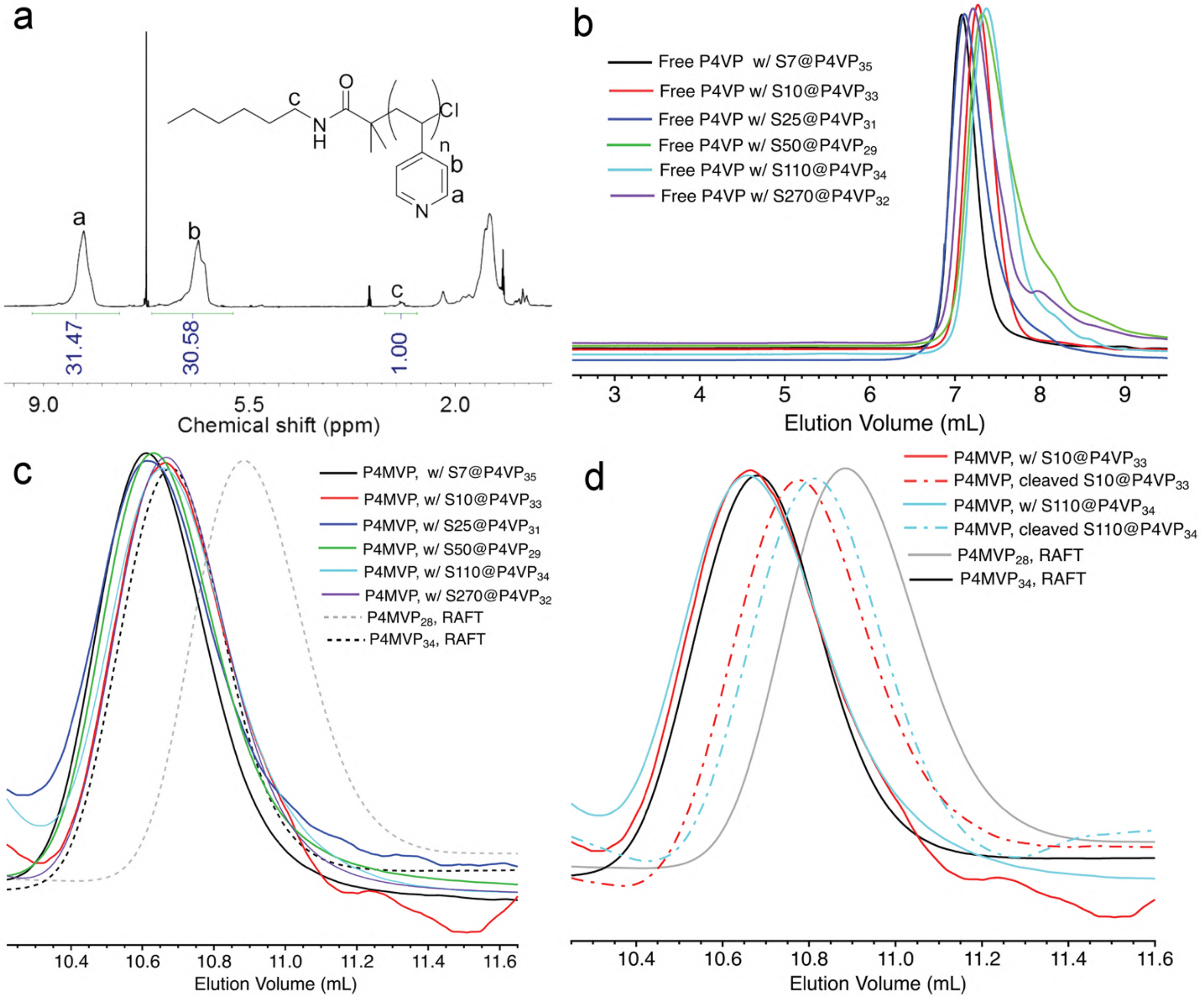

Figure 6. The well-defined polymer brushes on model SiO_2@P4VP as characterized by NMR and GPC.

The DP of free P4VP can be estimated using NMR by comparing the integration of peaks from P4VP to that from the initiator. **a**, As an example, NMR spectrum of free P4VP synthesized in the same batch as S25-P4VP31 is shown. When the number of protons from peak *c* is set to 1, there are 31 protons from peak *a* or peak *b*, suggesting a DP of 31 for the P4VP brushes. **b-c**, GPC traces of free P4VPs synthesized in the same batch with individual model SiO_2@P4VP are measured in either DMF (**b**; with 0.02M ammonium acetate) or water (**c**; after P4VP is converted to P4MVP). All of them have very close peak positions with small PDIs (see Table 1 in main text). Two well-defined P4VP chains, $P4VP_{28}$ and $P4VP_{32}$, respectively, synthesized by reversible addition-fragmentation chain transfer (RAFT) polymerization,[14] are quaternized to their P4MVP forms and measured as controls to show the well-defined and nearly identical size of free polymers grown simultaneously with individual SiO_2@P4VP (**c**), or cleaved polymer brushes from the SiO_2@P4VP nanoparticles (**d**).

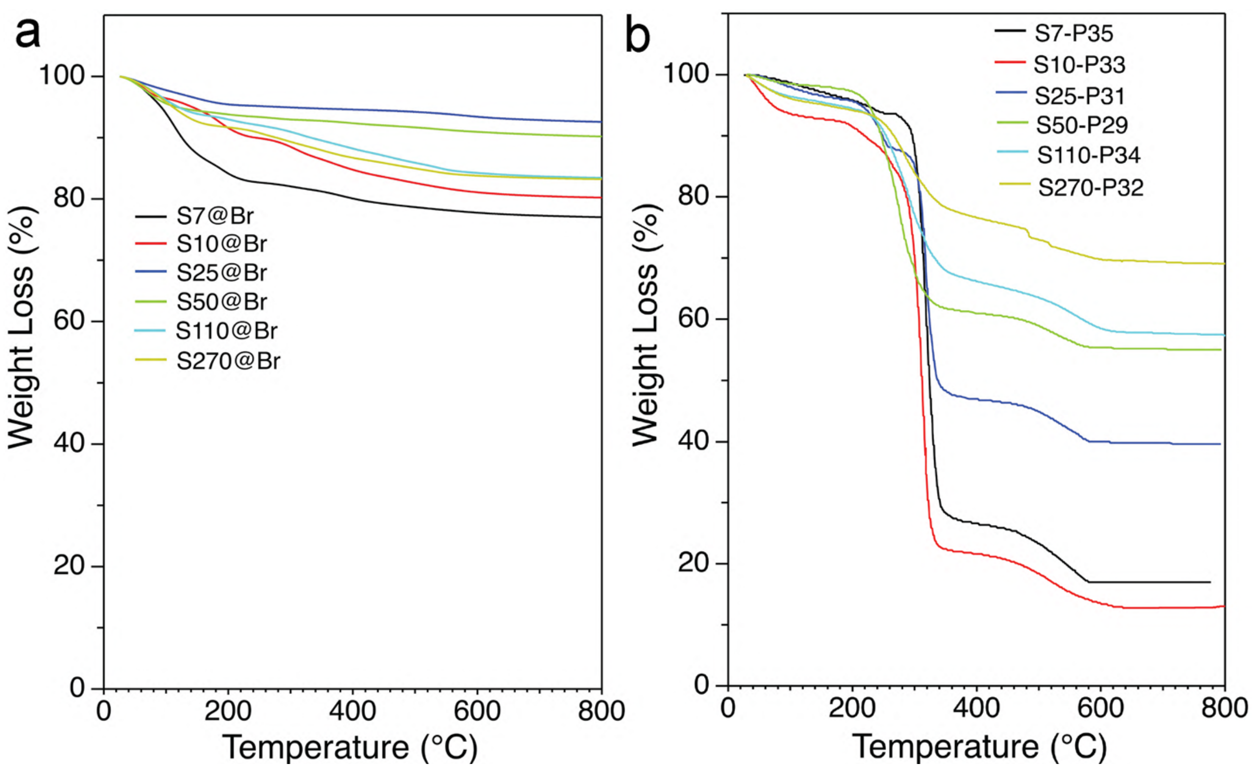

Figure 7. TGA of silica nanospheres before and after SI-ATRP of polymer brushes to prepare model NPPBs.
a, SiO_2@Br. **b**, NPPBs.

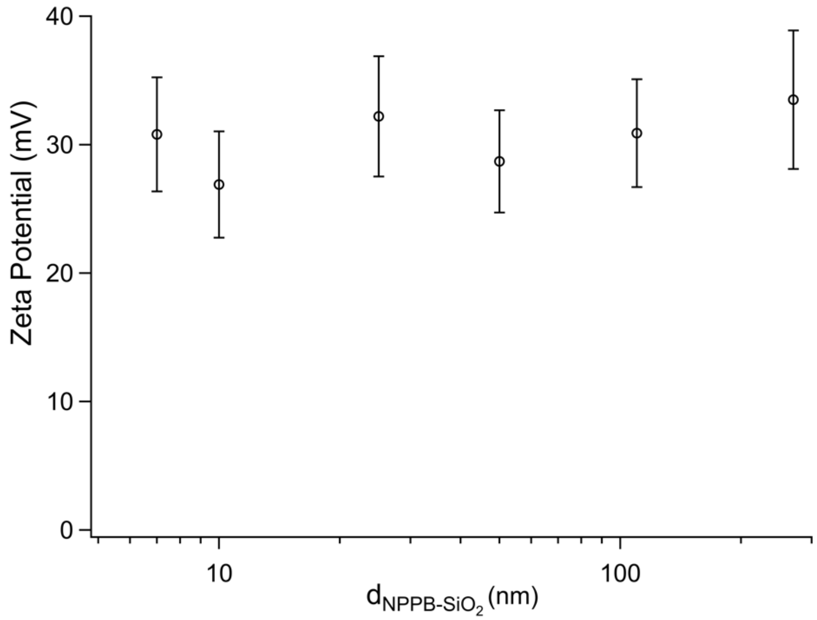

Figure 8. Zeta potentials of model NPPBs.
The zeta potentials of all model NPPBs are similar, i.e., 30±4 mV when measured in 10 mM NaCl at pH 7, reflecting their similarly-sized P4MVP brushes. Each sample was measured independently 3 times. Error bars = Standard Deviation (n=3).

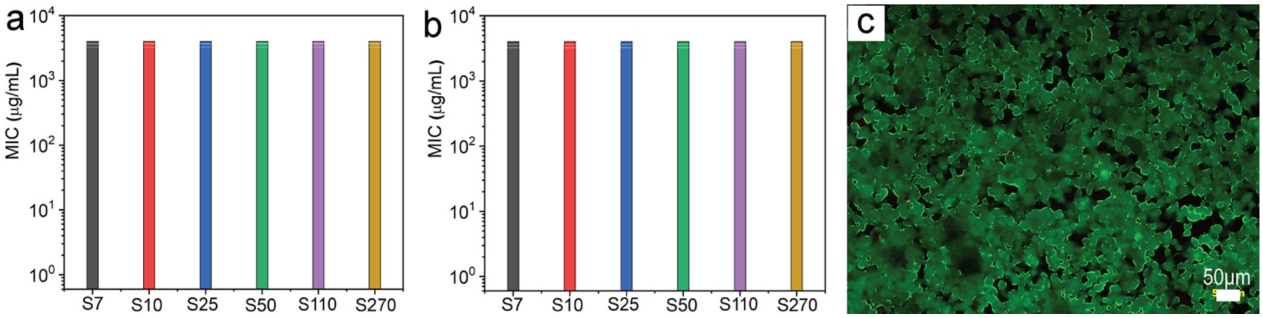

Figure 9. Bare silica nanopsheres without polymer brushes show no antimicrobial activity or cytotoxicity.

a, MIC assay against *E. coli*. **b**, MIC assay against *S. aureus*. and **c**, an example of the live/dead staining assay of HEK-293 cells incubated with S270 at 512 μg/mL. Similar snapshots of live HEK-293 cells as shown in **c** are consistent across all confocal microscopy pictures taken in different experiments (n=5).

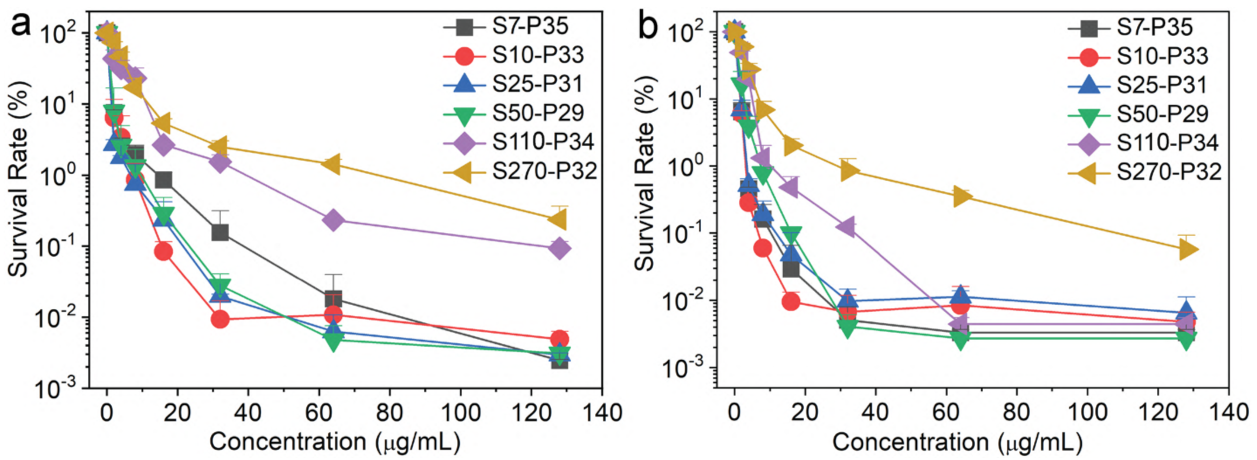

Figure 10. MBC assays of model NPPBs reveal nanoparticle size-dependent bactericidal activity.
a, against *E. coli*. **b**, against *S. aureus*. Error bars = Standard Deviation (n=8).

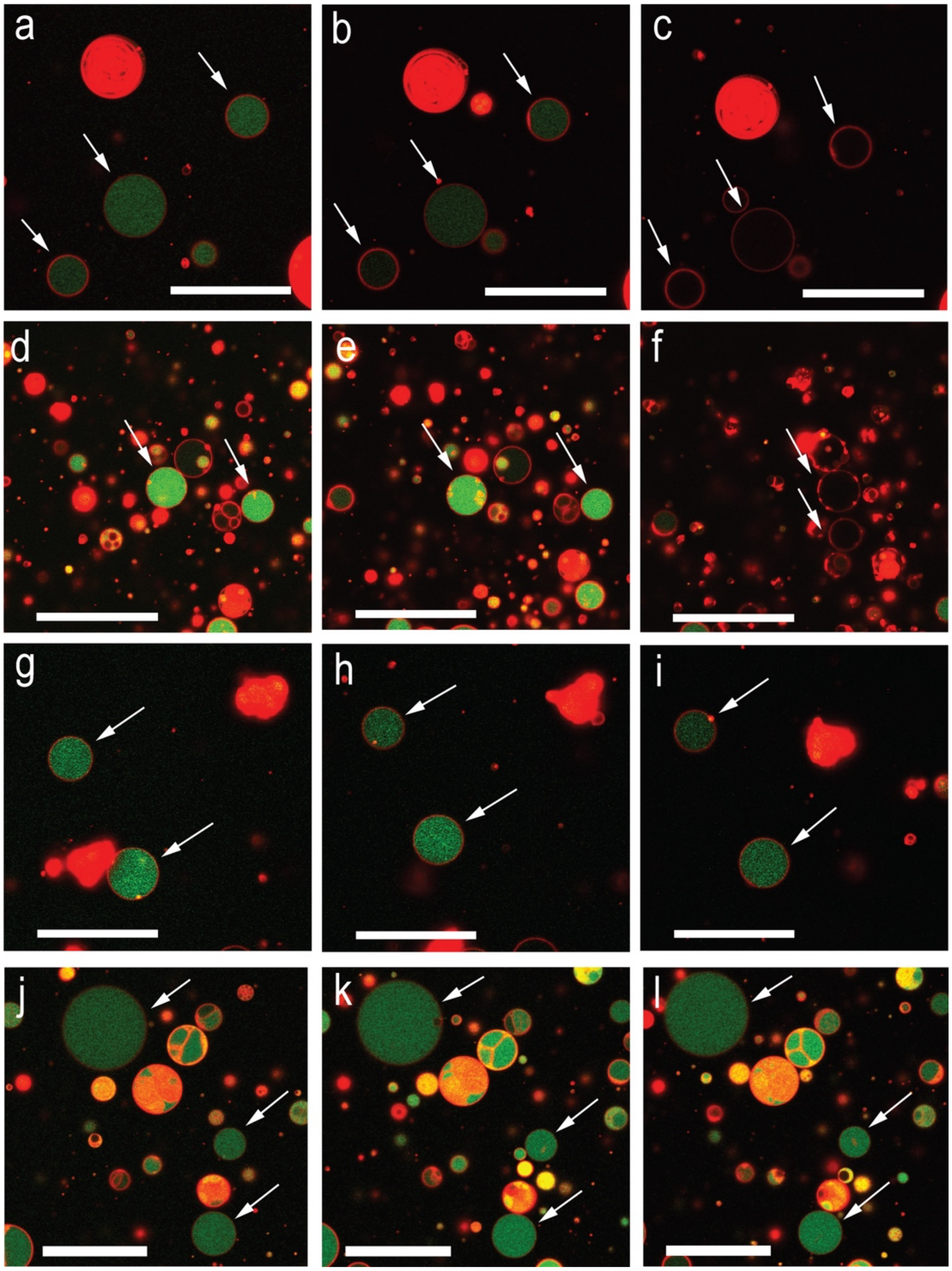

Figure 11. Snapshots examples of dye leakage assays that reveal NPPBs remodel bacteria- and mammalian cell-mimicking GUVs in different modes depending on both the intrinsic curvature of membrane lipids and nanoparticle size.

a-i, Time-lapse confocal microscopy pictures of bacteria-mimicking GUVs (DOPG/DOPE=20/80) interacting with S10-P33 (**a-c**), S25-P31 (**d-f**), and S270-P32 (**g-i**) for different times (**a**, **d**, **g**: 0 s; **b**, **e**, **h**: 300 s; **c**, **f**, **i**: 600 s) after introducing the NPPBs at t=0 s. **j-l**, Time-lapse confocal microscopy pictures of mammalian cell-mimicking GUVs (DOPG/DOPC=20/80) interacting with S10-P33 for 0 s (**j**), 300 s (**k**) and 600 s (**l**). The GUVs are loaded with fluorescein (green) and the GUV membranes are labeled with 18:1 Liss Rhod PE (red). Arrows are used to help track the GUVs as they move over time during the measurement. Scale bar: 50 μm.

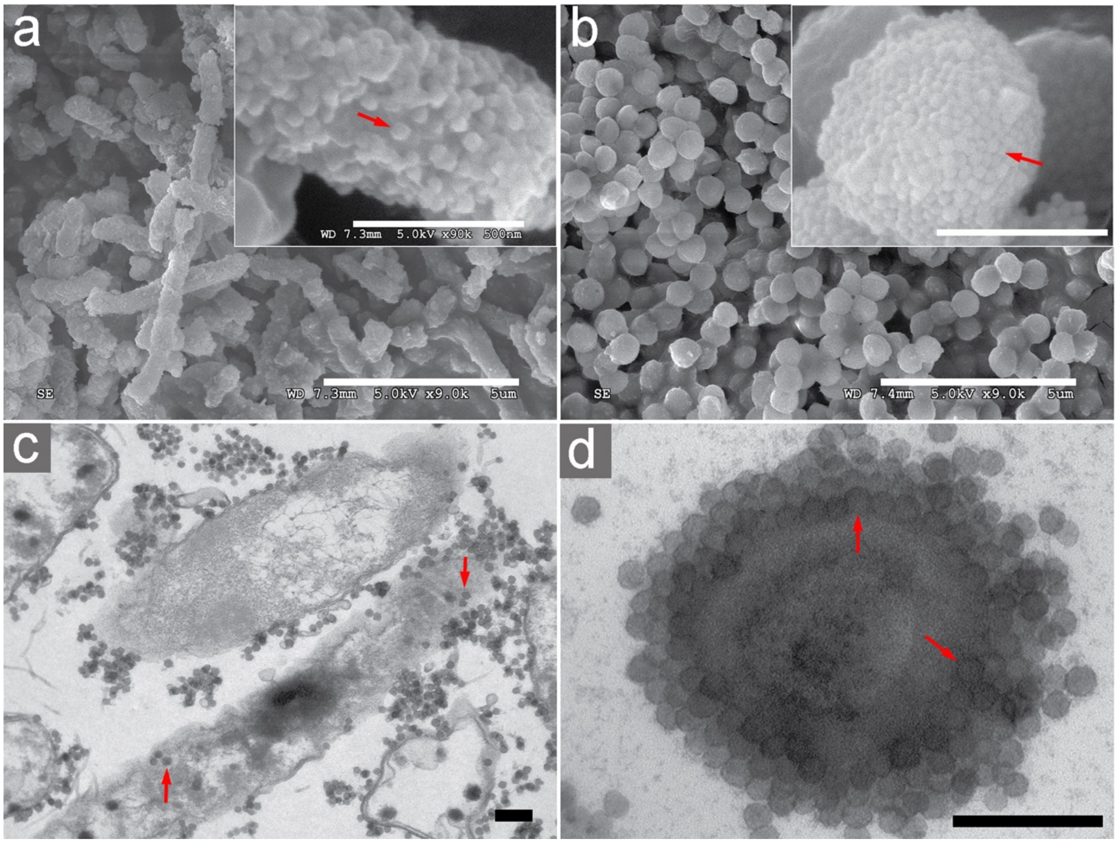

Figure 12. The targeting and disruption of bacterial membranes by NPPBs is independent on bacterial membrane potential.

Carbonyl cyanide *m*-chlorophenyl hydrazone (CCCP) was used to dissipate the membrane potential and pH gradient across bacterial membranes before the bacteria were treated by NPPBs. The SEM of **a**, *E. coli* and **b**, *S. aureus* at low (scale bar: 5 µm) and high resolutions (insets; scale bar: 500 nm), respectively, after incubated with S25-P31 show that the NPPBs target and anchor onto bacterial membranes in a similar manner as that observed for the nascent bacteria unexposed to CCCP (see Figure 3 in main text). While the fragmentation of *E. coli* is obvious (**a**), no clear morphological change is observed for *S. aureus* (**b**). Cross-sectional TEM of **c**, *E. coli* (scale bar: 200 nm) and **d**, *S. aureus* (scale bar: 200 nm) treated by CCCP, followed by S25-P31 incubation and OsO_4 staining reveal that the NPPBs completely obliviate bacterial plasma membranes, with some of the NPPBs (pointed by red arrows) even encroach into the cytoplasmic space of the bacteria. Similar SEM and TEM micrographs as shown in **a-b** and **c-d**, respectively, are consistent across all pictures of individual samples taken in different experiments (n=6).

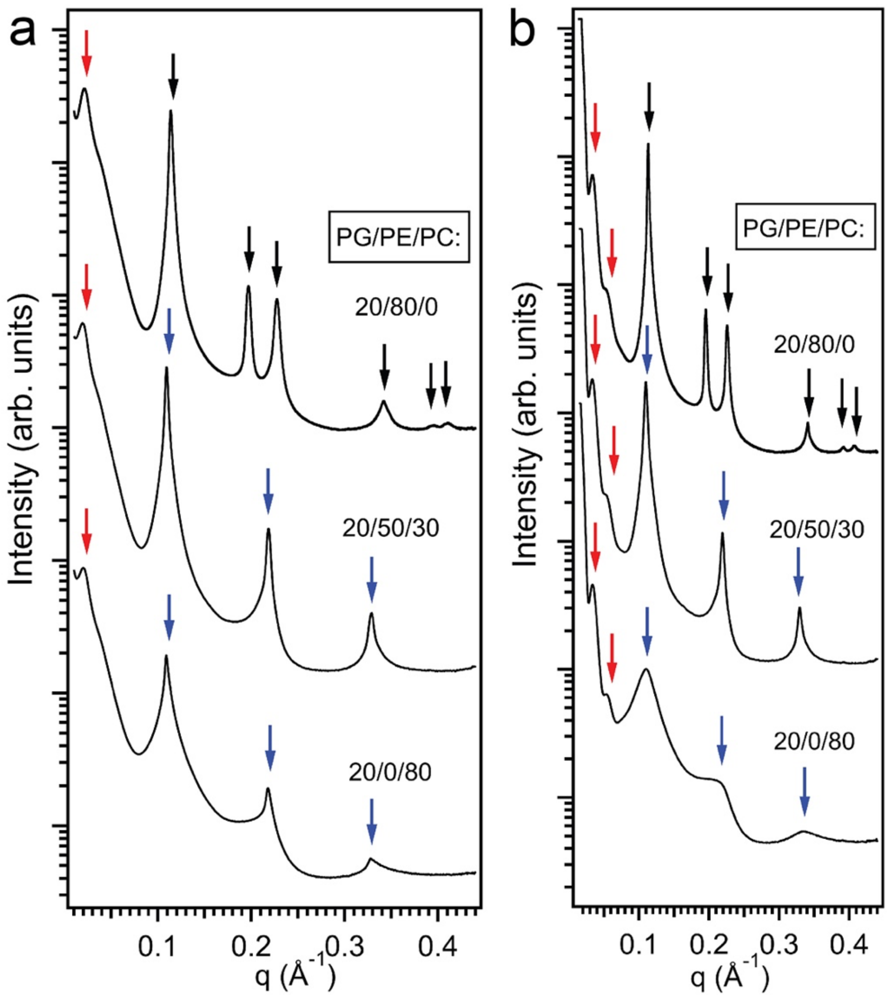

Figure 13. SAXS reveals that the formation of pores in model membranes encountered with small NPPBs (d_{silica}≤50 nm) depends highly on their content of negative-intrinsic-curvature lipid. Example SAXS traces of model membranes comprised of a tertiary mixture of DOPG, DOPE, and DOPC with a constant membrane charge density (i.e., 20% DOPG) but different PE/PC ratios remodeled by S7-P35 (**a**) and S25-P31 (**b**), respectively, show that formation of the 2D H_{II} phase only occurs for the bacteria-mimicking membrane with a high PE content (i.e., ~80%). The scatterings from silica nanoparticles are marked by red arrows, while the scatterings from the lamellar and 2D H_{II} membrane phases are marked by the blue and black arrows, respectively. See Table 3 in the main text for details about the peak identifications.

4 References

1. Davis TM, Snyder MA, Krohn JE, Tsapatsis M. Nanoparticles in lysine-silica sols. *Chem. Mater.* **18**, 5814-5816 (2006).

2. Yokoi T, Sakamoto Y, Terasaki O, Kubota Y, Okubo T, Tatsumi T. Periodic arrangement of silica nanospheres assisted by amino acids. *J. Am. Chem. Soc.* **128**, 13664-13665 (2006).

3. Yokoi T*, et al.* Mechanism of Formation of Uniform-Sized Silica Nanospheres Catalyzed by Basic Amino Acids. *Chem. Mater.* **21**, 3719-3729 (2009).

4. Hartlen KD, Athanasopoulos APT, Kitaev V. Facile preparation of highly monodisperse small silica spheres (15 to > 200 nm) suitable for colloidal templating and formation of ordered arrays. *Langmuir* **24**, 1714-1720 (2008).

5. Xia YN, Gates B, Yin YD, Lu Y. Monodispersed colloidal spheres: Old materials with new applications. *Adv. Mater.* **12**, 693-713 (2000).

6. Stöber W, Fink A, Bohn E. Controlled Growth of Monodisperse Silica Spheres in the Micron Size Range. *J. Colloid Interface Sci.* **26**, 62 (1968).

7. Sun YB, Ding XB, Zheng ZH, Cheng X, Hu XH, Peng YX. Surface initiated ATRP in the synthesis of iron oxide/polystyrene core/shell nanoparticles. *Eur. Polym. J.* **43**, 762-772 (2007).

8. Ohno K, Akashi T, Huang Y, Tsujii Y. Surface-Initiated Living Radical Polymerization from Narrowly Size-Distributed Silica Nanoparticles of Diameters Less Than 100 nm. *Macromolecules* **43**, 8805-8812 (2010).

9. Ranjan R, Brittain WJ. Combination of living radical polymerization and click chemistry for surface modification. *Macromolecules* **40**, 6217-6223 (2007).

10. von Werne T, Patten TE. Atom Transfer Radical Polymerization from Nanoparticles: A Tool for the Preparation of Well-Defined Hybrid Nanostructures and for Understanding the Chemistry of Controlled/"Living" Radical Polymerizations from Surfaces. *J. Am. Chem. Soc.* **123**, 7497-7505 (2001).

11. Li DX, He Q, Cui Y, Li JB. Fabrication of pH-Responsive Nanocomposites of Gold Nanoparticles/Poly(4-vinylpyridine). *Chem. Mater.* **19**, 412-417 (2007).

12. Kuang L*, et al.* Spontaneous Microalgae Dewatering Directed by Retrievable, Recyclable, and Reusable Nanoparticle-Pinched Polymer Brushes. *Chem. Mater.* **31**, 4657-4672 (2019).

13. Pietrasik J, Tsarevsky NV. Synthesis of basic molecular brushes: ATRP of 4-vinylpyridine in organic media. *Eur. Polym. J.* **46**, 2333-2340 (2010).

14. Jiang YJ, Zheng W, Kuang LJ, Ma HR, Liang HJ. Hydrophilic Phage-Mimicking Membrane Active Antimicrobials Reveal Nanostructure-Dependent Activity and Selectivity. *ACS Infect. Dis.* **3**, 676-687 (2017).
